# Critical Review of Read‐Across Potential in Testing for Endocrine‐Related Effects in Vertebrate Ecological Receptors

**DOI:** 10.1002/etc.4682

**Published:** 2020-03-04

**Authors:** Margaret E. McArdle, Elaine L. Freeman, Jane P. Staveley, Lisa S. Ortego, Katherine K. Coady, Lennart Weltje, Arnd Weyers, James R. Wheeler, Audrey J. Bone

**Affiliations:** ^1^ Exponent Maynard Massachusetts USA; ^2^ Exponent Washington DC USA; ^3^ Exponent Chapel Hill North Carolina USA; ^4^ Environmental Safety, Bayer CropScience Chesterfield Missouri USA; ^5^ Toxicology and Environmental Research and Consulting, Dow Chemical Midland Michigan USA; ^6^ BASF SE, Agricultural Solutions‐Ecotoxicology Limburgerhof Germany; ^7^ Crop Science Division Bayer Monheim am Rhein Germany; ^8^ Shell Health, Shell International B.V. The Netherlands

**Keywords:** Endocrine‐disrupting chemicals, Regulatory testing, Read‐across, Adverse outcome pathway, Predictive toxicology

## Abstract

Recent regulatory testing programs have been designed to evaluate whether a chemical has the potential to interact with the endocrine system and could cause adverse effects. Some endocrine pathways are highly conserved among vertebrates, providing a potential to extrapolate data generated for one vertebrate taxonomic group to others (i.e., biological read‐across). To assess the potential for biological read‐across, we reviewed tools and approaches that support species extrapolation for fish, amphibians, birds, and reptiles. For each of the estrogen, androgen, thyroid, and steroidogenesis (EATS) pathways, we considered the pathway conservation across species and the responses of endocrine‐sensitive endpoints. The available data show a high degree of confidence in the conservation of the hypothalamus–pituitary–gonadal axis between fish and mammals and the hypothalamus–pituitary–thyroid axis between amphibians and mammals. Comparatively, there is less empirical evidence for the conservation of other EATS pathways between other taxonomic groups, but this may be due to limited data. Although more information on sensitive pathways and endpoints would be useful, current developments in the use of molecular target sequencing similarity tools and thoughtful application of the adverse outcome pathway concept show promise for further advancement of read‐across approaches for testing EATS pathways in vertebrate ecological receptors. *Environ Toxicol Chem* 2020;39:739–753. © 2020 The Authors. *Environmental Toxicology and Chemistry* published by Wiley Periodicals, Inc. on behalf of SETAC.

## INTRODUCTION

Understanding the potential of chemicals to interact with the endocrine system of humans and wildlife has become a matter of public concern, regulatory focus, and research activity in recent years (Bergman et al. 2013; Leopold et al. 2017). Internationally recognized and harmonized test guidelines exist to evaluate whether a chemical has the potential to interact with the endocrine system in various organisms and to assess indirect and direct endocrine adverse effects (Organisation for Economic Co‐operation and Development 2012a; Japanese Ministry of the Environment 2019; US Environmental Protection Agency 2019a). However, animal toxicity testing is costly and time consuming. Given the impracticality of using animal tests to assess the large number of chemicals in commerce on multiple types of organisms, and the global initiative toward replacement, reduction, and refinement of animal testing (Russell and Burch 1959; De Wolf et al. 2007), alternative approaches are required. Methods based on biological read‐across for species extrapolation may be advantageous in assessing taxonomic susceptibility and prioritizing further testing. This is particularly relevant for endocrine active substances, where a concern may be driven by an endocrine mode of action.

Read‐across has several definitions and applications. Chemical read‐across is based on the concept that structurally similar chemicals are likely to have similar physicochemical and toxicological properties and is used to predict the environmental fate or effects of chemicals of the same or similar class (European Chemicals Agency 2017). This method has been used in the Organisation for Economic Co‐operation and Development (OECD) Screening Information Data Set program, the US Environmental Protection Agency (USEPA) High Production Volume Chemicals program, and the European Union Registration, Evaluation, Authorisation and Restriction of Chemicals program to fill data gaps for chemical hazard assessment. Biological read‐across is based on conserved biological aspects across taxonomic groups including receptor targets, physiology, shared life‐history traits, and so on, that can be used to predict responses in other taxonomic groups based on these conserved features.

Research on endocrine systems in vertebrates has largely focused on the estrogen, androgen, thyroid, and steroidogenesis (EATS) pathways in the hypothalamus–pituitary–gonadal (HPG) and the hypothalamus–pituitary–thyroid (HPT) axes. Thus the focus of the present study was on the HPG and HPT pathways, where most of the available data for biological read‐across exists. Other pathways (e.g., the hypothalamus–pituitary–adrenocortical axis, the somatotropic axis, the retinoid signaling pathway, the vitamin D signaling pathway, and the peroxisome proliferator activated receptor [PPAR]) are of emerging interest but are not discussed in the present study.

Endocrine pathways in most invertebrate species have not been well studied, and there are a vast number of species with a wide diversity of endocrine control pathways for growth, development, and reproduction; thus, it should not be assumed that pathways found in vertebrates are conserved in invertebrates (Coady et al. 2017). Given these limitations, our study did not include invertebrate species, but there may be good opportunity for biological read‐across in the future when invertebrate endocrine systems are better understood.

With the above considerations, the objective of our study was to evaluate the scientific support for biological read‐across in endocrine evaluations. We focused specifically on biological read‐across for vertebrate species examining the EATS pathways within the HPG and HPT axes.

## REVIEW OF BIOLOGICAL READ‐ACROSS

### General approach

Biological read‐across approaches predict toxicity from one taxonomic group to another based on the conservation of elements (receptors, enzymes, proteins) that drive the biological response. Therefore, understanding this conservation and identifying its presence (or absence) among vertebrates is critical. Conserved biological responses are often identified at the molecular level, but linking these responses to an adverse effect on an individual, let alone on populations, can be challenging. This is important because population‐level impacts are the basis for ecological hazard and risk assessments, and regulatory actions are based on these assessments.

A biological response to a specific chemical challenge can be described by the mode of action, but this may or may not describe an adverse outcome resulting from the challenge. Adverse outcome pathways (AOPs) describe biological changes from a molecular initiating event through biological processes called key events but also postulate an observable adverse outcome that results from these events. The adverse outcome of interest is a biological change, such as effects on survival, growth, development, or reproduction, relevant to both population‐based effects and risk assessment (Ankley et al. 2010). For instance, the biological response of the blockage of estradiol production can lead to reduced vitellogenin (VTG) production in egg‐bearing vertebrates (e.g., fish), which can lead to oocyte atresia and reduced fecundity (Ankley et al. 2010; Kramer et al. 2011) in individual organisms, potentially reducing the population of fish depending on the magnitude of the upstream effect and compensatory mechanisms in the fish population. The AOP approach takes existing knowledge of molecular initiating events and the array of intermediate key events across all levels of biological organization (molecular, cellular, tissue, and organ) that lead to an adverse effect on the individual or population. The AOP approach is extremely useful in read‐across, due to the identification of molecular initiating events and key events that may be common to different taxa. The growing availability of high‐throughput (HTP) screening approaches provides necessary mechanistic information to support AOPs. Individual AOPs can be combined into AOP networks through shared key event nodes. The OECD and other scientific groups have provided strategies, principles, and best practices for developing AOPs (Organisation for Economic Co‐operation and Development 2013; Villeneuve et al. 2014a, 2014b; Gray 2017).

This type of thinking has caused a shift in the type of testing being considered for regulatory ecotoxicology from whole‐animal (in vivo) toxicity testing relying on apical endpoints to hypothesis‐driven approaches. Such hypothesis‐driven approaches have merit and are being championed in research and development practices and some regulatory initiatives (European Chemicals Agency et al. 2018). The in vivo approach is labor intensive and expensive, has high animal use, and thus is impractical for testing the tens of thousands of chemicals in use or coming to market. The hypothesis‐driven approaches that may ultimately replace or at least specifically direct in vivo testing include in silico (e.g., quantitative structure–activity relationships [QSARs], 3D‐receptor modeling, and other computational approaches), in vitro (e.g., HTP and high‐content screening assays), and short‐term in vivo tests with pathway endpoints (e.g., ‘omics).

Current technologies that allow identification of protein sequences and molecular targets are providing information to underpin the long‐held assumption that phylogenetically similar organisms have similar responses to chemical exposures (see Rand‐Weaver et al. [2013] for an example phylogenetic tree that illustrates this concept). To investigate these relationships, the USEPA has developed an online tool (Sequence Alignment to Predict Across Species Susceptibility [SeqAPASS]) to quantitatively assess protein sequence and structural similarity across taxonomic groups (LaLone et al. 2016). Identifying an effect at the molecular level, and establishing links to the effects in the individual organism and ultimately the populations of organism, is the basis for the utility of the AOP concept. All these concepts and technologies are broadening our capabilities for understanding where biological read‐across can be applied.

### Surrogacy

The concept of biological read‐across is closely related to that of surrogacy, which has been used since the earliest days of toxicology and ecotoxicology. In toxicology, testing mammalian species such as rodents is regularly used to predict responses in humans. In recent years, as molecular biology tools have advanced and greater understanding of the molecular underpinnings of biology have revealed similarities across taxa, other ecological species have become of greater interest. In particular, *Danio rerio* (zebrafish) have also been used as a model for the study of human diseases and the discovery and development of human drugs (Zon 1999; Kari et al. 2007; Best and Alderton 2008; Chakraborty et al. 2009). There many advantages to their use: ease of culture, rapid embryonic development, visibility of organ development due to transparent embryos and juveniles, and access to the zebrafish fully sequenced genome. Most importantly, they are providing a deepening understanding of similarities to humans in molecular mechanisms of development (Kari et al. 2007). In addition, the National Institutes of Health supports research on the function or regulation of a zebrafish gene or its ortholog from humans, to investigate human development and the causes of developmental diseases.

In ecotoxicology, representative organisms of different taxa and trophic levels are tested. The results are assumed to cover toxicity to other taxonomic groups within that taxon or trophic level through conservative testing conditions (like maintaining concentrations over time or testing preferably sensitive life stages) or assessment factors. The typical suite of testing to evaluate aquatic ecological effects may include toxicity tests with representative fish, invertebrates, algae, and aquatic plants, and the screening of ecological effects on terrestrial organisms may include ecotoxicological studies on birds, earthworms, insects, and terrestrial plants (as well as mammals from the toxicology data set). The species commonly used in testing were originally selected based on ease of culturing or handling, availability, and commercial importance (Celander et al. 2011). The inherent assumption in the use of representative organisms is that other phylogenetically related organisms will respond similarly to toxicants; thus, for example, toxicity data for one freshwater fish species is used to represent phylogenetically similar fish species. The USEPA has developed a software tool, Web‐based Interspecies Correlation Estimation (Web‐ICE; US Environmental Protection Agency 2019b), to estimate acute toxicity to aquatic and wildlife species for which test data are absent based on the known toxicity to tested (surrogate) species that are phylogenetically related.

In assessing risk for reptiles, surrogacy is particularly important. Toxicological data are scarce for reptiles because current regulatory schemes do not require toxicity testing with reptiles, and thus standard laboratory protocols for reptiles do not exist. However, due to their phylogenetic proximity to birds, it may be possible to use existing avian data to estimate toxicity to reptiles. This is the approach currently taken in registration of crop protection chemicals in the United States. To explore this approach, Weir et al. (2015) investigated statistical relationships between reptile and bird acute toxicity data for a limited number of pesticides. Acute data consisted of 96‐h median lethal dose (LD50) values for any reptile species and for any avian species (13 chemical pairs). Correlation analysis was used to determine whether any significant relationships existed between the limited reptile and bird toxicity data. A highly significant relationship between reptile and avian toxicity was found when only definitive LD50s were used (8 chemical pairs). No significant relationship was observed when all available data for the chemical pairs were used; however, 5 chemical pairs had undefined LD50s due to lack of toxicity at the tested concentration ranges. This suggests avian data could be used to predict reptile toxicity; however, some pesticides were clearly more toxic to birds than reptiles and vice versa. For instance, reptiles appear to be more sensitive to pyrethroids than birds, whereas birds appear to be more sensitive to brodifacoum than reptiles (Weir et al. 2015). Field metabolic rates and food intake rates are much lower in poikilothermic reptiles than in birds, which markedly reduces dietary exposure to chemicals and offers an additional margin of safety when using avian hazard data as a surrogate for reptilian data (Nagy 1987). More toxicity data for reptiles are needed to evaluate predictive relationships with birds and establish chemical class‐specific or mode of action–based predictive relationships between the 2 or with other vertebrate classes (Weir et al. 2015).

Amphibians actually require 2 types of surrogates, due to their life history characteristics and associated exposure in either water (eggs and larvae) or on land (metamorphosized juveniles and adults). Fish appear to be good surrogates for aquatic life stages of amphibians because on average they are somewhat more sensitive; some chemicals demonstrating higher chronic sensitivity in fish include 17β‐estradiol, dichlorvos, and 4‐tert‐octylphenol (Weltje et al. 2013; Weltje and Wheeler 2015). Data on terrestrial life stages are scarce, but the available oral acute studies show that birds or mammals may be good surrogates because on average they are somewhat more sensitive (Crane et al. 2016). The dermal exposure of terrestrial life stages is an exposure route not typically covered in bird and mammal studies, but Weltje et al. (2017) developed a method for estimating lethal overspray values based on acute fish and fish bioconcentration factor data.

### Approaches for species extrapolation

#### Approaches developed using pharmaceutical data and concepts

Approaches have been developed in the past decade to leverage the extensive body of mammalian pharmacokinetic data to predict potential risks to fish from pharmaceuticals. The efficacy of these approaches is fundamentally based on the conservation of molecular targets between mammals/humans and fish. Gunnarsson et al. (2008) found a high degree of conservation of pharmaceutical targets (not specifically endocrine system targets) across taxa, particularly with the zebrafish (*D. rerio*), which exhibited 86% conservation. Some approaches have simply assumed that 100% homology exists between human and fish molecular targets, but more sophisticated approaches have recently been developed that use DNA sequences and domain binding analyses of molecular targets among humans and fish. Once a target has been identified in fish, potency should be determined from biochemical (e.g., receptor binding) and physiological responses.

Concluding that fish are not so biochemically different from mammals, Huggett et al. (2003) developed an approach, the theoretical fish plasma model, that compares the human therapeutic plasma concentration of a pharmaceutical with a predicted fish plasma concentration using a predicted or measured environmental concentration and an estimate of bioconcentration using the fish blood–water partitioning coefficient derived from the octanol–water partition coefficient (log *K*_OW_). The fish plasma concentration is based on steady‐state conditions, and thus is theoretical. If the theoretical fish plasma concentration approaches or exceeds the human therapeutic plasma concentration of a pharmaceutical, then that drug may pose a risk to fish. This approach assumes that the same toxicokinetics exist in both humans and fish and that the equilibrium partitioning theory can be used to predict uptake and fish plasma concentrations for all pharmaceuticals; both of these assumptions are not always true.

Several authors have promoted use of the fish plasma model approach to prioritize pharmaceuticals for further study but noted some key limitations (Fick et al. 2010; Schreiber et al. 2011; Roos et al. 2012; Caldwell et al. 2014). Fick et al. (2010) noted that ionized chemicals in fish plasma predicted by the fish plasma model are likely overestimated and that the model would benefit from a better understanding of other factors beyond lipophilicity (predicted by log *K*_OW_) that affect bioconcentration, such as plasma protein binding, metabolism, and tissue and organ partitioning. Schreiber et al. (2011) also noted the importance of factors other than lipophilicity, such as pH, in predicting fish plasma concentrations and environmental concentrations of drugs. Furthermore, Schreiber et al. (2011) suggested that concentrations lower than the human therapeutic concentration may be harmful to fish given the possibility of differing target sensitivity across species and the potential for a drug to interact with unintended targets.

Rand‐Weaver et al. (2013) argued that even though the fish plasma model is widely accepted, additional work is needed to fully validate it. To validate the fish plasma model, Margiotta‐Casaluci et al. (2014) exposed the fathead minnow (*Pimephales promelas*) to the antidepressant drug fluoxetine for 28 d to produce plasma concentrations that bracketed human therapeutic plasma concentrations and linked anxiety‐like endpoints (measured by tracking swimming behavior as fish explored different areas of the tank) observed in exposed fish. Fluoxetine is a selective serotonin reuptake inhibitor (SSRI), and the targets for this drug are highly conserved between humans and fish. Fish plasma concentrations of fluoxetine and its metabolite caused a related effect only when they were similar or higher than the upper value of human therapeutic plasma doses. Although the effective fish plasma concentrations did not completely overlap with the human therapeutic plasma concentration range, humans affected by generalized anxiety also show a similar range in sensitivity. Similar to the study design of Margiotta‐Casaluci et al. (2014), Valenti et al. (2012) exposed *P. promelas* to sertraline (an SSRI) and found decreased serotonin reuptake transporter binding in the brain and reduced shelter‐seeking behavior (a sign of reduced anxiety) in exposed adult male fish. Levels of sertraline measured in blood plasma of exposed fish exceeded therapeutic levels for humans based on the fish plasma model when adjustments for pH and the distribution constant (log *D*) were made. Thus, the work by Margiotta‐Casaluci et al. (2014) and Valenti et al. (2012) were the first studies to validate the fish plasma model.

Similar to the fish plasma model, Kostich and Lazorchak (2008) estimated concentrations of each active pharmaceutical ingredient (API) in wastewater and compared it with the peak concentration freely dissolved in human plasma after administration of a minimum therapeutic dose. (This assumes similar cellular potency across mammals and aquatic organisms.) Out of the top 50 APIs expected in wastewater, 11 had hazard quotients greater than 1; all but 2 (estradiol and atorvastatin) had hazard quotients less than 10. Their model used several simplifying assumptions, such as external (wastewater) concentrations equaled internal (extracellular fluid) concentrations and no dilution with receiving water. These factors make the model likely to substantially overestimate exposure; however, the model did not include exposure from sediment or diet, in which concentrations may be substantially higher, and thus the approach may lead to underestimations of exposure in that regard. In addition, as with the fish plasma model, their model is also based on pharmacological responses in human models and not whether these concentrations may lead to ecologically relevant effects in fish (such as reproduction, growth, and survival).

Another approach to predicting chronic toxicity data for fish for untested pharmaceuticals is using mammalian acute‐to‐therapeutic ratios (ATRs), which are similar to acute‐to‐chronic ratios (ACRs) in aquatic toxicity (Berninger and Brooks 2010). Berninger and Brooks (2010) found a statistically significant relationship between mammalian ATRs and fish ACRs based on chronic fish response to pharmaceuticals with similar therapeutic modes of action; thus, high mammalian ATRs, which are readily available, may be a promising way to identify drug compounds with a high potential to cause chronic toxicity in fish.

The approaches just discussed pertain to pharmaceuticals designed to be biologically active through specific modes of action. Other chemicals, such as industrial chemicals, were not designed to be biologically active; thus, they tend not to exhibit selective toxicity; however, the framework just discussed for pharmaceuticals can be applied to nonpharmaceuticals whose toxicity occurs via known specific pathways, such as endocrine pathways.

#### Approaches using in vitro and in silico data and concepts

Although some approaches leverage the wealth of available pharmaceutical data for read‐across, in vivo toxicity data for nonmammalian vertebrates are generally lacking for large numbers of nonpesticide chemicals in commerce. In silico, HTP and high‐content screening approaches based on mammalian models and associated AOPs can provide information on molecular initiating events that can be used to prioritize testing in other vertebrate taxa (Perkins et al. 2013). Data from HTP in vitro tests are being generated for extensive sets of chemicals of environmental and commercial interest, with the expectation that in vitro assay data could ultimately be used to predict adverse effects of chemical exposures in vivo.

**Figure 1:**
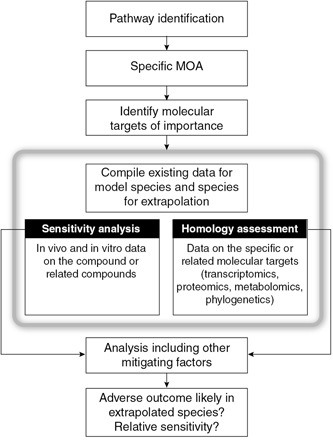
Conceptual framework for species extrapolations. Figure adapted from Celander et al. (2011). MOA = mode of action.

Next‐generation approaches have expanded our insights into AOPs (including molecular initiating events, key events, and population‐based effects) in ways that in vivo animal toxicity testing could not. Celander et al. (2011) have proposed a generalized cross‐species extrapolation approach (Figure 1). The approach begins with identifying the molecular pathway affected by a chemical or group of similar chemicals. The AOPs specific to those chemicals are then used to identify molecular targets of interest and molecular initiating events, such as receptor binding or enzyme inhibition. These data can often be collected from the large body of mammalian‐based HTP data available from testing programs such as the USEPA's ToxCast and the Endocrine Disruptor Screening Program in the 21st Century (EDSP21; Dix et al. 2007; US Environmental Protection Agency 2011). Data are then compiled for tested species (e.g., mammals) and species of interest (e.g., fish, reptiles, birds, or amphibians). Homology among the test species and species of interest is studied using target sequencing similarity and other ‘omics data. Recently, computational tools have been developed to search large databases of molecular sequences and structural data to assess molecular target similarity across taxonomic groups (LaLone et al. 2013a, 2013b, 2014). However, it may not always be clear that the appropriate target(s) have been identified, and such uncertainties are typically addressed in the use of these approaches in risk assessment.

LaLone et al. (2013a) used molecular target information for a given chemical (e.g., drug or pesticide) with a known mode of action from publicly available databases to identify homolog candidate protein sequences in nontarget species for a wide range of taxonomic groups. The percentage similarity of the protein sequences in nontarget species was calculated and given a score based on whether it was statistically similar to the target species protein sequence. Another database was used to determine the number of conserved domains in common to assess the quality of the protein alignment between the target and the nontarget species. As long as the number of conserved domains between the protein of the nontarget and target species was at least one, the sequence was included. Protein orthologs were then identified using reciprocal best‐hit analyses, and the percentage similarity was determined. These were then rank‐ordered and compared with toxicity data for aquatic species to see whether the computational approach for determining species sensitivity based on molecular target similarity aligned with the empirical toxicity data.

Other approaches have been taken to compare human ligand‐binding sites with other vertebrate protein orthologs. McRobb et al. (2014) selected 28 human proteins that had known ligand‐binding pockets and at least one known ligand (e.g., PPAR gamma [PPARγ], estrogen receptor alpha [ERα], estrogen receptor beta [ERβ], thyroid receptor alpha, and thyroid receptor beta) and searched databases for protein orthologs in fish species (*D. rerio* [zebrafish], *P. promelas* [fathead minnow], and *Takifugu rubripes* [Japanese pufferfish]) and frog species (*Xenopus laevis* [African clawed frog] and *Xenopus tropicalis* [Western clawed frog]). Analyses of sequence alignment found that the full sequence of most of the human proteins had 60 to 70% similarity in aquatic vertebrate orthologs. Variations in the full sequences among species were apparent for several human proteins (e.g., ERs [ERα, ERβ], androgen receptor [AR], and glucocorticoid receptor). Analyses of ligand–pocket similarities found much higher conservation than full sequences for most proteins. For instance, the full sequence similarity to ERα ranged between 53 and 77% for the 5 aquatic vertebrates, whereas the binding pocket sequence similarities to ERα ranged between 98 and 100% for the 5 aquatic vertebrates. Based on the 28 target proteins assessed, the Western clawed frog had the highest conservation with human protein binding pockets (21 target proteins), followed by zebrafish and Japanese pufferfish (19 target proteins), African clawed frog (18 target proteins), and fathead minnow (7 target proteins). The genome of the fathead minnow has not been fully sequenced, which may explain why fewer fathead minnow protein binding pockets were similar to human protein binding pockets. Two protein targets, PPARγ and corticotropin‐releasing factor receptor 1 (CRF_1_R), showed lower sequence similarity in the binding sites than in the full sequence. Those authors noted that CFR_1_R generally exhibits a greater degree of binding pocket sequence variability. The pregnane X receptor and constitutive androstane receptor, which function to regulate metabolism, transport, and excretion of both xenobiotics and endogenous compounds, were not well conserved in fish and frogs compared with humans; therefore, biological read‐across for these pathways may not be robust to predict biological responses in these taxa.

Rider et al. (2009a) compared ligand binding by full‐length human ERα and fathead minnow Erα for 12 chemicals using both a whole‐cell assay and a cell‐free assay. In general, the human and fish data were comparable within the same system. In another study, similar concordance was found between human ERα binding affinity and bird (Japanese quail, *Coturnix japonica*), reptile (alligator, *Alligator mississippiensis*), amphibian (Japanese giant salamander, *Andrias japonicus*), and fish (fathead minnow); however, the comparison was done using a structurally limited set of chemicals (17β‐estradiol, ethinylestradiol, dihydroxytestosterone, and corticosterone; Rider et al. 2009b). In comparing the ability of contaminants from Lake Apopka, Florida (USA), to bind with human ERα and alligator Erα, Rider et al. (2010) concluded that mammalian ERs are appropriate surrogates for other species in in vitro tests used for screening chemicals for estrogenicity, because there were no examples of a chemical binding with moderate or high affinity to the ERs of one species and not binding at all to the ERs of another species. In the analysis of Ankley et al. (2016), data on transactivation assays with ERα ligand‐binding domain from species representing 4 vertebrate classes showed good agreement between data from humans, birds, reptiles, and amphibians.

Matthews et al. (2000) compared ERα competitive binding for human, mouse, chicken, green anole, and rainbow trout and found good agreement for the higher affinity steroid‐like chemicals but less similarity when comparing chemicals with lower affinity for ERα. Dang (2010) conducted a review of data on ER binding affinities for mammals and fish for 65 chemicals, concluding that ER binding in one vertebrate species or one subtype of ER could be extrapolated to other species or subtypes of ERs for most chemicals and that differences in relative binding affinity were more attributable to interlaboratory differences than species differences. In further work on the ERs, Dang et al. (2011) concluded, based on a review of data for 90 chemicals from transactivation assays with 14 types of human cells and 5 types of fish cells, that there were no significant differences between them.

Doering et al. (2019) compared in vitro aromatase inhibition to evaluate the potential sensitivity of 18 phylogenetically diverse species of freshwater fish to the aromatase inhibitor fadrozole. Aromatase inhibition in response to fadrozole ranged more than 52‐fold among fish species. However, relative potency estimates across a range of substances (Hinfray et al. 2006; Beijer et al. 2018; Doering et al. 2019) compared with mammalian‐based measures from the interactive Chemical Safety for Sustainability ToxCast Dashboard indicated that only 2 substances differed by more than 1 order of magnitude. This comparison indicates that there is potential for the mammalian‐based assays to be potentially useful to predict relative potency in fish.

Conceptually, these types of information could be taken together to predict the susceptibility of the species of interest to a group of similar chemicals with the same molecular initiating events or key events (same AOPs). Chemical groups could then be ranked according to species sensitivity to inform priorities for in vivo testing. The AOPs have been proposed as a way to prioritize chemicals for ecotoxicity testing and environmental assessments (Ankley et al. 2010; Caldwell et al. 2014; Tollefsen et al. 2014). It must be kept in mind that in vitro assays do not adequately account for toxicokinetic and toxicodynamic interactions via absorption, distribution, metabolism, and excretion (ADME) or tissue‐ and species‐specific effects and are not amenable to many chemical substances (Gray 2017). Thus, these assays may have limited utility for predicting adverse in vivo responses and assessing risks (Matthews et al. 2002). A significant challenge for biological read‐across approaches for vertebrates relates to differences in species sensitivity; however, the use of available toxicokinetic data allows for assessments of how well mammalian data can be used for other vertebrates for a given AOP (Villeneuve et al. 2014a, 2014b). This is because toxicokinetics affect how a chemical reaches the target and how potent that chemical will be to the organism (Celander et al. 2011). Toxicokinetic data are important for determining whether the chemical has the potential to interact with the target, whether there is metabolic activation or de‐activation, and whether there are species‐specific toxicokinetic or toxicodynamic responses to the chemical (sensitivity and potency).

However, tools are continuously being developed and refined to improve the predictivity of in vitro data. Translating values obtained from in vitro assays into estimates of in vivo outcomes is a complex process involving mathematical modeling and increasingly complex test systems. Physiologically based pharmacokinetic models predict the relationship between the internal dose of a substance and the external exposure (in diet, water, etc.) to that substance by accounting for ADME processes. In vitro to in vivo extrapolation models allow in vitro data to be extrapolated to estimate corresponding in vivo effects.

## BIOLOGICAL READ‐ACROSS FOR EATS

For the evaluation of endocrine‐active substances in vertebrate wildlife, the concepts of biological read‐across provide useful constructs because the characterization of chemicals as endocrine disruptors is based on specific AOPs (e.g., the chemical interacts with a receptor in the endocrine system or affects a neuro‐endocrine pathway in the organism that leads to an adverse effect). This is consistent with the World Health Organization/International Programme on Chemical Safety definition of an endocrine disruptor as “an exogenous substance or mixture that alters function(s) of the endocrine system and, consequently, causes adverse health effects in an intact organism, or its progeny, or (sub)populations” (Damstra et al. 2002). The major pathways discussed in our review were selected according to the OECD Detailed Review Paper (Organisation for Economic Co‐operation and Development 2012b), based on 1) evidence in the literature of susceptibility to disruption by environmental chemicals with potential for adverse outcomes, and 2) availability of standardized assays or assays with procedures sufficiently developed to be standardized and validated.

Some AOPs for endocrine activity via the HPG and HPT pathways have been developed (Ankley et al. 2005, 2010; Miller et al. 2009) and are discussed in the following sections, *HPG axis* and *HPT axis*. Researchers have also developed AOPs for other endocrine pathways (e.g., hypothalamus–pituitary–adrenal, somatotropic, and PPAR) that can be found on the AOP‐Wiki website (Society for the Advancement of Adverse Outcome Pathways 2019).

### HPG axis

The primary role of the HPG axis in adult vertebrates is controlling reproduction; however, it also controls differentiation of sex‐specific phenotypes during early development (Norris and Carr 2013). Secondarily, the HPG axis in vertebrates plays crucial roles in other systems, such as metabolism, growth, immune function, and cardiovascular function (Norris and Carr 2013). The HPG axis is highly conserved among vertebrates (Norris and Carr 2013; Gray 2017). For instance, gonadotropins, follicle‐stimulating hormone, and luteinizing hormone are found in most vertebrates (Sower et al. 2009; Levavi‐Sivan et al. 2010; Norris and Carr 2013), and their primary function, which is to stimulate development of reproductive organs and gametes, is also similar among vertebrates. In addition, sex steroids are also conserved among vertebrates. Gray (2017) reported that key events and molecular initiating events in the estrogen and androgen signaling pathways are highly conserved among species from fish to humans. Interference with the HPG axis can include agonism and antagonism of estrogens, androgens, and gestagens and interference with the genesis of sex steroids.

Ankley et al. (2005, 2010) developed AOPs for ER‐mediated reduction of VTG leading to impaired reproduction in fish. Ankley et al. (2010) also described aromatase inhibition that leads to reduced estradiol, which in turn reduces VTG and leads to impaired reproduction in fish. A quantitative version of this AOP includes a computational model of the steps from aromatase through VTG production, an oocyte dynamics growth model, and a population model (Conolly et al. 2017). Ankley et al. (2010) described a third endocrine‐related AOP, the AR‐mediated reduction of gonadotropin production, which leads to a reduction in testosterone that leads to a reduction in estradiol (because estradiol is produced from testosterone). The reduction of estradiol leads to reduced VTG levels, which can lead to impaired reproduction in fish. Although the molecular initiating events differed for all 3 of these endocrine AOPs, the adverse outcome, impaired reproduction, was the same. Ankley et al. (2016) evaluated the literature on VTG induction for 7 chemicals that have been determined to be positive in the mammalian uterotrophic assay: ethinylestradiol, diethylstilbestrol, genistein, bisphenol A, nonylphenol, methoxychlor, and *o,p*′‐DDT. The responses in fish, amphibians, reptiles, and birds were compared with that in the uterotrophic assay. Overall, there was good qualitative agreement between positive results in the uterotrophic assay and in the nonmammalian vertebrates, with the limited data available.

Estradiol is the most common estrogen in vertebrates (Lange et al. 2003). The naturally occurring estrogen, 17β‐estradiol, and the structure and specificity of the ERs are remarkably similar in all vertebrates (Kloas et al. 2009). It has been suggested that this specificity of ERs between vertebrates indicates that all vertebrates, including amphibians and fish, should be suitable for assessing estrogenic and anti‐estrogenic endocrine‐mediated effects (Kloas et al. 2009). However, predominant androgens and gestagens differ among mammals, amphibians, reptiles, and fish. The principal androgens in humans are testosterone and dihydrotestosterone, but 11‐ketotestosterone is the primary androgen in fish (Organisation for Economic Co‐operation and Development 2012b). Testosterone and dihydrotestosterone found in anurans are the same as in all higher vertebrates (Kloas and Lutz 2006). In mammals and amphibians, progesterone is a key gestagen, but fish appear to have other, more dominant gestagens; the major progestin in teleosts is 17,20β‐dihydroxypregn‐4‐en‐3‐one (17,20β‐P) or, in certain teleost species, 17,20β,21‐trihydroxy‐pregn‐4‐en‐3‐one (17,20β,21‐P; Scott et al. 2010). In addition to hormone homology, steroid receptors, such as the ERs, appear to be conserved among vertebrates; however, the number of ER subtypes can differ. Two forms (ERα and ERβ) are found in mammals, whereas ray‐finned fish species (Actinopterygii), the most commonly used fish species in endocrine research, have at least 3 distinct subtypes, including Erα, Erβ‐I, and Erβ‐II (Hawkins et al. 2000; Nelson and Habibi 2013). Rainbow trout has 4 Er forms: Erα1 (*esr1a*), Erα2 (*esr1b*), Erβ‐1 (*esr2a*), and Erβ‐2 (*esr2b*; Nagler et al. 2007). The fish Erα is homologous to the mammalian ERα. Fish Erβ‐II shares the most identity with mammalian ERβ‐II. A mammalian homolog of the fish Erβ‐I has not been identified. Nelson and Habibi (2013) reviewed ER function and regulation in fish and proposed that the observed species variation in the homologous regulation of the ER subtypes is due in part to sex, dose regimen, and reproductive stage, and recommended that these factors be considered in studies. The differences in tissue distributions of Erα, Erβ‐1, and Erβ‐2 in the brain and estrogen target tissues suggest that they have different functions in these tissues (Hawkins et al. 2000). Relatively little information is available on the functional significance of the different Er isoforms in fish.

The specifics of the pathways (e.g., target tissues and effects) diverge, and a wide array of reproductive strategies are employed by vertebrates. For instance, estradiol induces the production of VTG, a precursor to egg yolk proteins, in fish and other oviparous vertebrates. However, VTG is absent in mammals and other nonoviparous vertebrates.

Interaction with the HPG axis has been well studied in fish (Giesy and Snyder 1998; Kime 1998). The focus of earlier work was on feminization in fish, because field evidence of this was mounting in the early 1990s in the United Kingdom (Matthiessen and Gibbs 1998). More recent research has uncovered endocrine‐related effects from other pathways, such as androgens and gestagens, in fish. Androgenic effects were observed in fish following exposure to trenbolone and its metabolites (Wilson et al. 2002). Anti‐androgenic effects, such as reduced sperm counts, intersex in male fish, and other effects, have also been reported (Kiparissis et al. 2003; Ankley et al. 2004; Panter et al. 2004; Kang et al. 2006; Katsiadaki et al. 2006; Zeilinger et al. 2009).

The HPG axis interactions in amphibians have also been well studied. However, most of the work has focused on sex determination with biomarkers including gonadal morphology and hormone levels or specific proteins (Linder et al. 2010). Exposing *X. laevis* to model androgens and anti‐androgens resulted in effects in both tadpole and adult stages (Bögi et al. 2002; Kloas 2002; Cevasco et al. 2008). Androgenic and anti‐androgenic–mediated effects on anurans might be more closely related to mammals, including humans, than to androgenic and anti‐androgenic–mediated effects on urodeles and fish, because anurans and mammals share the same predominant androgens (testosterone and dihydrotestosterone), and the predominant androgen in urodeles and fish is 11‐ketotestosterone (Kloas et al. 2009). Contraceptive gestagens appear to have a large effect on oviduct development in amphibians (Kvarnryd et al. 2011).

Few studies have been conducted with endocrine‐active substances on the HPG axis in reptiles (Grillitsch and Schiesari 2010). Florida researchers discovered that alligators in Lake Apopka, which was contaminated with agricultural runoff, sewage effluent, and a spill of DDT and dicofol, exhibited various reproductive effects, including abnormal levels of sex steroids and gonad morphology (Matter et al. 1998). Impaired reproductive success led to dramatic reductions in the population of Lake Apopka alligators. A proposed mechanism for the reproductive effects was early exposure of juvenile alligators to estrogenic contaminants, but the precise endocrine mode of action was not established. Rider et al. (2010) assessed the ability of the contaminants in Lake Apopka to bind to the alligator Erα in comparison with human ERα. They found that some of the chemicals had a slightly higher affinity for alligator Erα, but the difference was striking only for *p,p*′‐dicofol. They were unable to connect the observed in vitro response to an organismal level response in the alligator, because data available on the sex reversal response to a dicofol mixture were only suggestive of endocrine activity. Furthermore, the dicofol mixture did not elicit any estrogenic effect in the rat uterotrophic assay.

The HPG axis interactions in birds have also been investigated. The HPG axis in birds is associated with territorial aggression (Wingfield 2012). Female–female nest pairing in herring gulls and skewed sex ratios with an overabundance of females were observed in areas contaminated with DDT (Colborn et al. 1993). These observations have been used as an example of estrogenic effects observed in wild populations of birds. Laboratory studies demonstrated that abnormal gonadal development in birds occurred following DDT exposure; however, direct evidence of causation is lacking, and such field observations may have alternative explanations, such as skewed ratios from higher male mortality or rapid expansion into new areas (Feyk and Giesy 1998). Research on the mechanism for eggshell thinning from DDT exposure suggests effects on prostaglandins, whose synthesis is mediated by progesterone (Feyk and Giesy 1998); however, mechanisms of DDT‐induced eggshell thinning appear to be quite complex, and more recent research suggests eggshell thinning can result from malformed shell glands and reduced carbonic anhydrase activity from exposure to DDT and other estrogenic compounds (Holm et al. 2006).

Ankley et al. (2016) considered 3 “tiers” of information to conduct a comparative evaluation across taxa of ERα as a molecular initiating event for perturbation of estrogen signaling pathways that result in adverse outcomes. The first tier, evaluation of structural conservation, was based on the likelihood that a chemical might react with an “unknown” target based on its structural similarity to that used for the human ERα‐based HTP assays. This is similar to the use of protein sequence data discussed in the section *Approaches using in vitro and in silico data and concepts*. In this analysis, SeqAPASS displayed comparable sequence similarity among vertebrate classes. The second tier considered ERα functional conservatism, as shown by in vitro competitive binding assays and transcriptional activation assays. The authors re‐analyzed data from other studies on these endpoints. The third tier of information was available data from in vivo assays. It was concluded that chemicals found to be moderately to highly estrogenic in binding and activation studies had similar results in mammalian and nonmammalian vertebrate binding and activation tests; however, chemicals with low estrogenic potency in mammalian in vitro tests may be missed as potential chemicals that interact with Erα in fish and reptiles (Ankley et al. 2016). Good agreement was found for chemicals with varying estrogenic potencies between in vivo mammalian testing results (the uterotrophic assay [US Environmental Protection Agency 2019a] was used as the mammalian test) and nonmammalian results (in vivo induction of VTG), but data were limited for reptiles and birds, and most nonmammalian tests used moderate to high estrogenic potencies (Ankley et al. 2016).

Ankley and Gray (2013) used data generated from the tier 1 EDSP (US Environmental Protection Agency 2019a) development and validation programs to compare responses of mammals and fish exposed to 12 chemicals known to interfere with the HPG axis. Most chemicals tested positive in the fish and at least one of the rat tier 1 EDSP assays; however, the fish short‐term reproduction assay (FSTRA) was best at detecting chemicals that act as steroidogenesis inhibitors, whereas the Hershberger and pubertal male tests were better at detecting AR activity. This suggests that 2 tests (FSTRA and pubertal male assay) could be used to provide full coverage of the HPG axis pathways of concern and serve as initial “gate keepers,” with negative results ruling out some chemicals for further testing or positive results directing confirmatory testing. Specifically with regard to the ERs, a number of investigators have found concordance between humans and fish for in vitro assays (see section *Approaches using in vitro and in silico data and concepts*).

### HPT axis

The primary function of the HPT axis in vertebrates is controlling growth, development, and metabolism (Zoeller et al. 2007), which is regulated by the production and control of thyroid hormones. The HPT axis is highly conserved among vertebrates (Zoeller and Tan 2007; Zoeller et al. 2007; Norris and Carr 2013). For instance, triiodothyronine (T3) and thyroxine (T4) are found in all vertebrates (Norris and Carr 2013), and their primary functions are also similar among vertebrates, which are to control growth, development, differentiation, and metabolism as well as roles in reproduction, molting, and thermogenesis. In addition, all vertebrates have thyroid receptors that are part of the superfamily of nuclear hormone receptors (Organisation for Economic Co‐operation and Development 2012b). However, in some teleost fish and amphibians, corticotropin‐releasing hormone releases thyroid‐stimulating hormone, not thyroid‐releasing hormone (TRH), as is the case for mammals. Also, growth hormone and prolactin in teleost fish and amphibians appear to be stimulated by TRH (Organisation for Economic Co‐operation and Development 2012b).

The central regulatory role of thyroid hormones in metamorphosis has been identified in anuran amphibians, such as *X. laevis*, and in flatfish, such as the flounder (Miwa and Inui 1987), and may play a wider role in larval–juvenile transitions in fish. Tail resorption in anuran amphibians provides a sensitive endpoint for testing morphological changes that can be based on changes in thyroid hormone levels (Pickford 2010).

Miller et al. (2009) used the AOP approach to describe how organic contaminants lower thyroid hormones and thus interfere with the HPT axis in humans. Given the medium to high degree of conservation of the HPT axis in vertebrates, the AOP of Miller et al. (2009) is likely applicable to nonmammalian vertebrates. In fact, Perkins et al. (2013) described multiple AOPs for thyroid interference in amphibians and fish that begin with various molecular initiating events but lead to reduced thyroid hormone (T3 and T4) levels that can lead to thyroid tumors and death.

Although many mechanisms of action are conserved among vertebrate taxa, differences in target tissues and effects of thyroid hormones are seen among vertebrates. For instance, prolactin plays an important role in the production of breast milk in mammals, but in fish, prolactin aids osmoregulation (Manzon 2002), and in amphibians, it plays a role in metamorphosis (Norris and Carr 2013). In addition, thyroid hormones are important for fish spawning behavior, metamorphosis of anurans and flatfish, and stream recognition during smoltification (the series of physiological changes whereby juvenile salmonid fish adapt from living in fresh water to living in seawater) in salmonid fish (Norris and Carr 2013). Thyroid hormones also play a critical role in avian development. In precocial birds, a rise in thyroid hormones accompanying hatching is implicated in the control of the transitions necessary after hatching, including muscle development, lung maturation and the switch from chorioallantoic to pulmonary respiration, yolk sac retraction, gut development, and induction of hepatic genes to accommodate the change in dietary energy source, initiation of thermoregulation, and the final stages of brain maturation as well as early posthatch imprinting behavior (De Groef et al. 2013).

Primary mechanisms of thyroid activity in rodent and amphibian models include inhibition of thyroid synthesis, increased metabolism and elimination of thyroid hormone, interference with serum transport proteins, effects on peripheral deiodination of thyroid hormones, thyroid hormone receptor agonism/antagonism, and altered central regulation of the thyroid axis or other modes of action (Pickford 2010).

Amphibians appear to be an important test species for HPT axis interactions. Johnson et al. (2017) stated that data for long‐term exposures and metamorphosis suggest that amphibians are more sensitive than fish and other vertebrates to compounds that affect the HPT axis, citing Kerby et al. (2010) and Veldhoen et al. (2006). Veldhoen et al. (2006) found that triclosan altered thyroid hormone–associated gene expression, increased hindlimb development, and decreased total body weight. However, an analysis by Pickford (2010) examined 27 chemicals for which appropriate amphibian assays were available for comparison with mammals. There was only one chemical, methoxychlor, for which there was evidence of thyroid activity in the amphibian metamorphosis assay but not in mammalian assays. The authors argue that there is no need for using amphibian testing in endocrine disruptor screening evaluations for thyroid effects if there are mammalian data because there is a high degree of conservation of modes of action on thyroid activity with the mammalian assays. For endocrine activity, especially thyroid activity, fish respond with a sensitivity similar to that of amphibians (mainly *X. laevis*; see Weltje et al. 2013), and making use of read‐across between mammalian‐based mechanistic data and amphibians is encouraged (Lagadic et al. 2019).

Although assays with amphibians may be considered useful in ecological risk assessment, and the utility of their unique metamorphosis process lends itself well to investigating effects on thyroid activity, amphibians do not appear to be more sensitive than fish (in general; Weltje et al. 2013) or mammals (with respect to thyroid effects; Pickford 2010).

### Confidence in biological read‐across for EATS

As stated above, considerable information is available regarding biological read‐across for endocrine‐active substances for the HPG and HPT axes. Based on current understanding, the level of confidence in the conservation of these pathways between fish, amphibians, and birds to those for mammals is presented in Table 1. Available data from existing testing protocols show a high degree of confidence in the conservation of the HPG axis between fish and mammals and the HPT axis between amphibians and mammals. Comparatively, there is less support for the conservation of the HPG axis between amphibians and mammals, the HPG axis between birds and mammals, the HPT axis between fish and mammals, and the HPT axis between birds and mammals. For steroidogenesis, too little is known; thus, confidence is low (Table 1). Although ER and AR homology provides some information about pathway conservation, data are needed to compare responses in estrogen and androgen biosynthesis between mammals, fish, birds, and amphibians. Recent work with SeqAPASS and ToxCast/Tox21 mammalian‐based HTP assays showed a high degree of conservation between human and vertebrate ARs, steroidogenesis enzymes, and thyroid receptors and enzymes for most vertebrate groups (LaLone et al. 2018). However, enzymes involved in glucocorticoid synthesis in some classes, such as Aves (birds) and Chondrichthyes (cartilaginous fishes), differed from human, and a 17β‐hydroxy‐steroid dehydrogenase differed between Lepidosauria (reptilian group) and Chondrichthyes compared with human (LaLone et al. 2018). These results for protein homology can be used as a line of evidence for pathway homology, but several factors (as discussed in the section *Considerations and Limitations for Applying Biological Read‐Across Approaches*) can influence susceptibility to a chemical. Data on endocrine pathway conservation for reptiles are limited. Although good agreement was found for chemicals with moderate to high estrogenic potencies between in vivo tier 1 mammalian uterotrophic assay and nonmammalian in vivo induction of VTG, the data were limited for reptiles (Ankley et al. 2016). Comparisons of mammalian and reptilian in vitro assays have shown that chemicals with low estrogenic potencies had higher affinities to reptilian ERα than mammalian ERα (Rider et al. 2009a, 2009b; Ankley et al. 2016). In addition, data on the conservation of HPT axis and steriodogenesis pathways in reptiles are also scant. Therefore, reptiles were not included in Table 1.

**Table 1:** Level of confidence in conservation of pathways between ecological receptors and mammals for hypothalamus–pituitary–gonadal (HPG) and hypothalamus–pituitary–thyroid (HPT) axes

Pathway/axis	Fish and mammals	Amphibians and mammals	Birds/reptiles and mammals
HPG axis	High	Medium	Medium
HPT axis	Medium	High	Medium
Steroidogenesis	Low	Low	Low

## CONSIDERATIONS AND LIMITATIONS FOR APPLYING BIOLOGICAL READ‐ACROSS APPROACHES

One of the key tenets of biological systems is the ability to maintain homeostasis. Endocrine systems interact with a variety of biological processes and have multiple feedback and control mechanisms. At this time, there are limited real‐life examples of population‐based effects for chemicals in the environment acting specifically through endocrine modes of action. Although a controlled exposure of an entire lake to a synthetic estrogen (17α‐ethinylestradiol) resulted in the collapse of the fathead minnow population within 3 yr (Kidd et al. 2007), other studies have found that populations of a cyprinid fish living in effluent‐contaminated river stretches where feminization is widespread were self‐sustaining (Hamilton et al. 2014).

Some researchers have claimed that increases in wildlife populations are tied to risk management practices that have restricted the use of persistent organic pollutants with endocrine activity (Bergman et al. 2013). The researchers state that as chemical exposures increased, populations declined and, conversely, as chemicals were removed from the market and as exposures declined, populations recovered. This logic would be reasonable if 1) the chemical exposures were documented; 2) the levels of exposure occurring were sufficient to impact the organisms; 3) the organism‐level impacts were manifested in population‐level impacts; and 4) other possible causes for population changes were adequately considered. However, this has not been adequately demonstrated, and knowledge gaps in one or more of these factors are evident even for 2 of the most well‐studied suspected endocrine disrupting pollutants, DDT and tributyltin (Lamb et al. 2014). In addition, Matthiessen et al. (2017) concluded that sufficiently reliable risk assessment of endocrine‐disrupting substances could be accomplished provided that data on environmental exposures, effects on sensitive species and life stages, including delayed effects, and effects at low concentrations are robust. We would suggest that, in addition, other potential causes of adverse effects must be considered to form definitive conclusions about endocrine‐active substances.

The AOP approach provides a useful framework for biological read‐across. However, AOPs are, in general, qualitative frameworks that currently appear to be most useful for hazard assessment, whereas quantitative approaches for predicting adverse apical effects are needed for risk assessment (Wheeler and Weltje 2015). There is work underway to develop quantitative AOPs that could have applications in regulatory frameworks (Conolly et al. 2017). Some AOP networks that connect AOPs with common elements to more realistically capture complex biological interactions leading to adverse outcomes are also being developed (Knapen et al. 2015). In applying AOPs, it is important to distinguish between key events and the relevance of those key events in terms of overall systemic toxicity and any adverse outcomes in the whole organism and population. For example, estrogen binding assays indicate the ability to bind to the receptor but in and of themselves do not indicate an adverse effect, or an effect that would be the basis of an endpoint for regulatory assessment. Effects on systemic toxicity and reproduction in the whole animal system must be evaluated. A recent publication discusses the importance and challenges of distinguishing between endocrine disruption and nonendocrine effects (Marty et al. 2018).

The QSAR and HTP approaches are promising tools for reducing animal use and selecting appropriate test models and endpoints for screening and testing chemicals for endocrine activity, but there are currently a number of limitations. At present, genomes for most species have not yet been fully sequenced, which limits target sequence similarity analyses. In addition, these methods only address cross‐species target similarities and do not account for other factors that influence toxicity. For one, life stage can have a large influence on the susceptibility of an organism to a toxicant. Reproductive strategies and other life history traits as well as species‐specific biology may also affect how chemicals exert toxicity across species. In addition, toxicokinetics are critical to understanding species sensitivity to toxicants (Celander et al. 2011; LaLone et al. 2014), but data necessary for parameterization of predictive toxicokinetic models may be lacking. Finally, functions of proteins may differ substantially across taxa. QSARs may provide insights into protein functions among vertebrate taxa (Celander et al. 2011); however, each QSAR model should be evaluated for the underlying data quality as well as for the extent to which the QSAR model can be applied to other substances, that is, which limits define the applicability domain (Organisation for Economic Co‐operation and Development 2005).

Computational models based on protein sequence and binding site similarity are promising approaches for cross‐species extrapolations, but relying solely on them may miss downstream effects in nontarget vertebrates or may overpredict the potential for interaction. For example, compounds activated through metabolism may be overlooked based on binding studies with parent compounds, and compounds inactivated through metabolism may be inaccurately associated with a biologically relevant effect. In addition, due to the complex nature of biological systems, the act of receptor binding will not always translate to a downstream adverse effect in vivo. Certain AOPs based on both in vitro and in vivo data for such compounds or related compounds will be needed to address potential oversights in using computational models for endocrine‐related read‐across approaches for test selection. In addition, conserved genes and hormones may have different roles among vertebrates, as discussed earlier in the example of the different roles that prolactin plays in mammals versus fish and birds (see the *HPT axis* section). Another example is the whole genome duplication event that occurred in the teleost fish lineage resulted in duplicate gene pairs (Glasauer and Neuhauss 2014). These gene pairs, referred to as paralogs, may consist of one gene that serves a homologous function and one inactive gene, a “sharing” of the function within the pair, or one gene taking on a new function. For example, most teleosts have one ERα gene (*esr1*) and 2 ERβ genes (*esr2a*, *esr2b*), which are all functional (Hsu and Sikora 2017). Understanding the biology of how these paralogs interact with ligands will determine how read‐across for fish at the gene and transcriptional level can occur. This phenomenon should be accounted for in any global validation efforts for read‐across methods.

Limitations of in vitro assays include the lack of pharmacokinetics in those systems, the lack of tissue‐ and species‐specific effects in those systems, and the lack of known molecular initiating events for some adverse effects (Gray 2017). Knowledge of specific targets and key events for a given endocrine pathway among vertebrates will be needed to fully understand the applicability of read‐across approaches for endocrine‐mediated effects. In addition, certain chemicals can be difficult to test in in vitro assays, such as those with high volatility and high hydrophobicity, or chemical substances of unknown or variable composition, complex reaction products, and biological materials. Testing these chemicals requires additional preparation steps to obtain accurate and reliable results, such as sterilization, preventing volatilitization, increasing solubility, and/or increasing homogeneity (Organisation for Economic Co‐operation and Development 2018). Analytical confirmation of the difficult‐to‐test chemicals may also be required at various steps of the testing procedure. The type of additional steps and analytical confirmation will depend on the properties of the chemical. Furthermore, it is important to identify the applicability domain in regard to physicochemical properties and chemical classes used to develop the assay (training sets/reference chemicals) and the limitations to what can be tested in the system (Organisation for Economic Co‐operation and Development 2014). An example of this type of identification is illustrated in publications describing the ToxCast chemical library. These describe the rationale for the chemical selection (e.g., drugs, antimicrobials, pesticides, food additives), selection criteria such as compound availability and dimethyl sulfoxide solubility, and the applicability domain within chemical space that will be relevant for any conclusions made from ToxCast data (Sipes et al. 2013; Richard et al. 2016).

Understanding how exposure influences response is critical. The biologically available exposure concentration is often not known with confidence for an in vitro assay. Even for in vivo studies in ecotoxicology, the actual internal dose is rarely known but is assumed based on the concentration in the exposure medium. Exposure differences between taxonomic groups in in vivo assays can result in different outcomes. For example, exposure of aquatic organisms through gills and skin can bypass metabolic pathways that might eliminate endocrine activity as would be observed in a mammalian species receiving an oral dose. Bisphenol A provides an example of the importance of ADME in the use of read‐across. In fathead minnows (waterborne exposure), this substance induces the production of VTG in male fish, but when female rats are orally exposed, even at very high doses, effects are minimal in the uterotrophic assay (Ankley et al. 2016). It is recommended that, for data‐rich chemicals, when the metabolism and toxicity of metabolites are known, these factors should be incorporated into extrapolations made using read‐across.

Validation schemes for read‐across approaches will also be needed before broad regulatory application. Validation is best accomplished using empirical data. Given the diversity and complexity of targets and pathways for endocrine‐active substances, empirical data will most certainly be lacking for some vertebrates and pathways; however, validation can begin with read‐across approaches for HPG interactions between humans and fish or HPT interactions between mammals and amphibians. Lessons learned from those validations can be applied to validation of read‐across approaches for other pathways and vertebrate groups. In addition, the degree of validation needed to provide sufficient confidence in a given read‐across approach has to be decided. Another complicating factor is the difference in validation schemes between regulatory authorities/validation bodies. Recognizing that global consensus will be needed if reduced vertebrate testing is to be realized, using a validation approach through an international organization, such as the OECD, is likely to be the most effective for gaining global acceptance. The OECD has developed guidance for validation and international acceptance of new or updated methods for hazard assessment (Organisation for Economic Co‐operation and Development 2005), and all OECD member countries as well as additional adherents are part of the OECD Mutual Acceptance of Data system.

In summary, applying biological read‐across approaches in selecting species for testing for endocrine‐related effects is quite promising. However, we do not yet fully comprehend the sensitive pathways or the interaction between pathways and endpoints for many species and chemical groups. In addition, evolving technologies such as HTP and high‐content screening assays and rapid DNA sequencing, which will be integral to supporting read‐across, will require standard methods and validation. Validation schemes for read‐across approaches for endocrine testing will also be important. Future research should focus on addressing these gaps in our understanding as well as standardizing and validating next‐generation testing and read‐across approaches instead of testing more species with traditional in vivo methods. In areas where confidence is lower for biological read‐across, descriptions of uncertainty should be incorporated as is done in risk assessments of chemicals with limited toxicological information.

## CONCLUSIONS

Given the work already conducted on pharmaceutical compounds with known (and intentional) modes of action, the most promising read‐across approaches for chemicals that interact with the HPG axis are currently between mammals and fish. These interactions and species differences have been fairly well studied. Fewer data exist for amphibians or birds, resulting in a medium degree of confidence in the concordance of HPG‐mediated responses between these groups and mammals. Although there can be some differences between the target tissues and hormonal mechanisms, the HPT axis is highly conserved among vertebrates. This results in higher confidence in read‐across potential between mammals and amphibians for this axis. Other axes and signaling pathways require additional research to identify species‐specific effects, modes of action, and AOPs. Future research should focus on filling in data gaps in current read‐across and computational approaches (e.g., sequencing genomes for many species, developing information for lesser known targets and key events among vertebrates, developing approaches to account for factors that influence toxicity, etc.), rather than developing tests on additional species.

In addition, important considerations for read‐across and computational approaches include potency, internal dosimetry, and exposure. In particular, exposure of aquatic organisms can be very different from exposure in mammals, because aquatic exposures can result in the lack of first‐pass metabolism, which can greatly reduce endocrine activity. Read‐across and computational approaches that rely on molecular or cellular assays may not work well for chemicals activated through metabolism. Moreover, conserved genes may have different roles among vertebrates (e.g., prolactin controls osmoregulation in fish and lactation in mammals) such that the adverse outcome may differ between species.

However, the long‐standing practice of using surrogate test species (e.g., biological read‐across) in ecotoxicology is supported by the observations of pathway conservation among vertebrates as well as the genetic homologies of receptors and proteins among vertebrates, which are becoming increasingly better defined. In addition, AOPs provide mechanistic support from key events at the receptor or genomic level through cellular‐ and organ‐based changes resulting in effects to whole organisms that, ultimately, may be linked to populations. Screening for chemicals based on key events in the AOPs and modes of action within and across taxa are promising approaches; however, the application of those key events in the larger context of internal dosimetry, potential exposure, potency, and whole‐animal effects needs to be used thoughtfully. Understanding interspecies and intraspecies similarities and variances will allow chemical modes of action and AOPs to be extrapolated more broadly across taxonomic groups (e.g., fish to reptile). This approach has particular utility for endocrine‐active substances when the main focus (and actually the definition of an endocrine‐active substance) is on the adverse outcome and chemical mode of action.

## Data Availability

Because this is a critical review, all data are from the references cited in the manuscript.
